# Anaemia Prevention Among Adolescent Girls in Lodhran, Punjab, Pakistan: A School-Based Weekly Iron-Folic Acid Supplementation Pilot Program

**DOI:** 10.3390/nu18142329

**Published:** 2026-07-16

**Authors:** Sumra Kureishy, Asim Shahzad Qureshi, Rakhshinda Ambreen, Shabina Raza, Marion L. Roche

**Affiliations:** 1 Nutrition International, Ottawa, ON K2P 2K3, Canada; sumrakureishy@gmail.com; 2 Nutrition International, Islamabad 44000, Pakistan; ashahzad@nutritionintl.org (A.S.Q.); rambreen@nutritionintl.org (R.A.); sraza@nutritionintl.org (S.R.)

**Keywords:** anaemia, adolescent girls, school-based supplementation

## Abstract

Background/Objectives: Pakistan has one of the highest burdens of adolescent anaemia in South Asia. A Weekly Iron-Folic Acid Supplementation (WIFAS) program for school-going adolescent girls aged 10–19 years was piloted in the Lodhran District, Punjab, Pakistan. Methods: A quantitative cross-sectional study was conducted among 750 girls, selected by multi-stage random cluster sampling from 50 public schools across Lodhran; qualitative data were generated through in-depth interviews and focus group discussions with 327 stakeholders. Quantitative data were analyzed using a chi-square test in SPSS. Qualitative data were analyzed in NVIVO using thematic analysis based on the Socio-Ecological Model, enabling environment and institutional systems, community engagement, behavior change and adolescent empowerment and agency. Coding was guided by overarching research questions of acceptability, feasibility and impact. Cases of anaemia averted were estimated using program data and outcome modeling. Qualitative findings were triangulated with quantitative results. Results: The program reached 30,506 girls and was estimated to have averted 5659 cases of anaemia. WIFAS coverage increased from 0% to 100%, with a high level of adherence (80%). Knowledge about anaemia, its signs/symptoms and preventative measures increased significantly at endline. Dietary practices showed positive changes, with significant increase in girls never skipping a meal. Stakeholders attributed success to the creation of an enabling environment through institutional alignment and multisectoral collaboration, culturally adapted, adolescent-friendly materials, and robust community mobilization that proactively addressed socio-cultural barriers. Conclusions: The school-based WIFAS program for reducing anaemia among adolescent girls was feasible and acceptable in a context where few nutrition services are available to girls.

## 1. Introduction

Adolescence is a critical development stage characterized by rapid physical, cognitive, and emotional growth [1,2]. This stage involves significant brain development and heightened nutritional demands that exceed both child and adult requirements, alongside evolving peer relationships and increased experimentation. Often referred to as a second critical window of opportunity for catch-up growth, adolescence offers a unique chance to address any nutritional deficits acquired during childhood, to optimize physical, cognitive, and metabolic health and to prevent productivity losses into adulthood [2,3].

Various risks to adolescent health and well-being persist, such as anaemia. As a global public health crisis, anaemia affects 33.7% of adolescent girls and women aged 15–49 years and 11.3% of adolescent boys and men aged 15–49 years worldwide [4]. Adolescent girls (10–19 years) are more affected by anaemia compared to their male counterparts [5,6,7,8]. Girls have a higher vulnerability to anaemia due to earlier onset of puberty, menstruation, and increased iron requirements and lower iron stores [8,9,10]. This is further exacerbated by gender disparities, which often limit their access to adequate nutritious food, education, and health services. Anaemia has multiple serious consequences for adolescent girls, including impaired physical capacity leading to fatigue and weakness, lower cognitive function affecting academic performance, higher vulnerability to infections, and reduced health [8,11,12,13,14,15].

South Asia is the epicenter of anaemia among adolescent girls and women aged 15–49 years (51.3%; 451 million) [16]. In this specific region, data are not available on the extent of anaemia caused by iron deficiency. Globally, it is estimated that up to 50% of anaemia is due to iron deficiency anaemia (IDA) [17]. IDA often represents an advanced stage of iron deficiency, but the causes of anaemia extend beyond iron alone; infections, hemoglobinopathies and deficiencies in vitamin A, riboflavin, folate, and vitamin B12 also contribute by impairing hemoglobin synthesis, restricting iron mobilization, or disrupting red blood cell production [18,19]. Within the region, Pakistan bears one of the heaviest burdens, with 54.7% of adolescent girls aged 10–19 years and 42.7% of women of reproductive age (WRA) experiencing anaemia [20]. Geographic and socioeconomic factors influencing adolescent anaemia include rural residence, low maternal education, and household poverty. According to the National Nutrition Survey 2018, the prevalence of anaemia is highest among girls belonging to the poorest wealth quintile (60.0%), living in rural areas (56.0%), and with mothers who had no education (55.6%) or only primary education (58.2%) compared to those in the richest quintile (48.7%), living in urban areas (52.6%), and whose mothers had higher education (48.0%).

Poor quality of adolescent diets further worsens this issue across Pakistan [20]. Although the majority of adolescents have a basic understanding of nutritious and healthy diets, their dietary practices are heavily reliant on staples such as grains, pulses, and dairy products [20]. Simultaneously, there is a growing preference for junk food such as chips, samosas, burgers and sugary carbonated drinks among adolescents, driven by low costs, easy access, variety, and taste. Barriers preventing uptake of healthier diets are weight and complexion concerns among girls, lack of flavor in home cooked meals among boys and cultural beliefs linked to hot/cold foods. These practices and preferences limit dietary diversity and compound the presence of nutritional deficiencies, especially anaemia.

The economic consequences of anaemia are substantial in Pakistan, with estimates suggesting anaemia in adolescent girls and women can cause losses in productivity and human capital of USD 595 million, equivalent to 0.2% of the gross national income (GNI) [21]. Furthermore, IDA is identified as the primary cause of lost disability-adjusted life years (DALYs) in adolescent girls aged 10–19, with 771,400 DALYs lost per year in Pakistan [22].

A Cochrane systematic review that informed the World Health Organization (WHO) guidelines demonstrated that intermittent iron supplementation reduced the risk of anaemia by 35% (RR 0.65, 95% CI 0.49–0.87) and increased hemoglobin by 5.19 g/L (95% CI 3.07–7.32) compared with a placebo, while producing comparable anaemia reductions to daily supplementation (RR 1.09, 95% CI 0.93–1.29) with a 59% lower risk of adverse side effects (RR 0.41, 95% CI 0.21–0.82) [23]. The studies included were predominantly conducted in school settings, often in contexts of higher school enrollment. No studies on adolescent girls aged 10–19 years from Pakistan were included in this review. The WHO guidelines recommend flexible, non-consecutive regimens (e.g., twice weekly, every other day) that may deliver iron alone or with other micronutrients for girls and women aged 15–49 years. However, the guidelines in Pakistan recommend a standardized regimen of Weekly Iron-Folic Acid Supplementation (WIFAS) with 60 mg elemental iron and 0.4 mg folic acid delivered through mass public health platforms (e.g., schools) for adolescents aged 15–19 years to prevent anaemia. Thus, adolescent girls are the primary subjects of our pilot program and evaluation [24,25].

Although the Adolescent Nutrition Strategy (2020–2025) existed, a practical blueprint for implementation was absent. To fill this operational void, the Ministry of National Health Services, Regulations and Coordination (MoNHSR&C), through partnership with Nutrition International (NI), under the Right Start Initiative, piloted a WIFAS program for school-going adolescent girls aged 10–19 years residing in the Lodhran District of Punjab province. Punjab was selected as the site for this pilot because it is home to most of Pakistan’s population and provides a representative snapshot of the lives of adolescent girls in the country [20]. The province performs better than the national average in several key nutritional indicators among adolescent girls, however stark regional inequalities exist within Punjab, with southern districts consistently faring worse (Table 1). These internal disparities make Punjab a strategic location for piloting an intervention that can be tested across a range of conditions and later scaled with necessary adaptations. Within Punjab, Lodhran was specifically chosen as the pilot district because it exemplifies the severe, overlapping nutritional challenges characteristic of the neglected southern region. In Lodhran, 10.2% of adolescent girls are underweight, including 2.0% who are severely underweight, while overweight and obesity concurrently affect 16.1% of girls. Short stature is prevalent in 22.7% of adolescent girls, with 6.3% experiencing severe stunting. Crucially, anaemia is even more widespread in Lodhran, affecting 56.6% of adolescent girls (Hb < 12 g/dL), a rate that surpasses both the provincial and the national averages. This triple threat of high anaemia, persistent undernutrition, and emerging overnutrition, combined with household food insecurity, underscored the urgent need for targeted adolescent nutrition interventions and made Lodhran an ideal setting in which to pilot and evaluate such an approach.

The pilot program was developed to demonstrate a localized delivery model for anaemia prevention among adolescent girls. To our knowledge, this is the first school-based, multisectoral delivery model for WIFAS to prevent anaemia among adolescent girls in Pakistan. The program was implemented between August 2019 and December 2021, in collaboration with the MoNHSR&C, the Integrated Reproductive Maternal, Newborn and Child Health and Nutrition Programme (IRMNCH&NP) in Punjab, the District Health Authority (DHA) in Lodhran, and the District Education Authority (DEA) in Lodhran.

An implementation evaluation was embedded within the pilot program to generate evidence on implementation realities, opportunities, challenges and lessons learnt to inform a future government-led scale-up. The overarching research question was “What is the feasibility of implementing WIFAS in schools as outlined in the Government of Pakistan guidelines?” The evaluation objective was to assess the acceptability and feasibility of a school-based, multisectoral WIFAS delivery model for adolescent girls in Lodhran, Punjab, Pakistan. Acceptability was based on perceptions of parents, teachers, and adolescents, and feasibility was assessed by the ability of the program to obtain high WIFAS coverage and consumption by students, successful delivery of WIFAS, and increased knowledge and awareness of anaemia and WIFAS among adolescent girls and their families. Feasibility of scaling up the model was discussed with stakeholders.

The pilot program aimed to prevent and reduce anaemia among school-going adolescent girls aged 10–19 across 50 public schools in Lodhran. It employed a multisectoral approach, partnering with district authorities and provincial government departments. The MoNHSR&C provided technical oversight, the IRMNCH&NP in Punjab provided overall coordination, the DHA acted as technical custodian and managed procurement, and the DEA served as the implementing partner within schools. Coordination was facilitated through District and Provincial Steering Committees. A multi-tiered framework of formal agreements governed this program, including a Memorandum of Understanding (MOU) between NI and the Economic Affairs Division at the national level, a Letter of Consensus with the IRMNCH&NP at the provincial level, and a joint MOU among the DHA and DEA to strengthen inter-departmental coordination.

The interventions included in the pilot program were WIFAS, and nutritionand anaemia awareness sessions. The WIFAS provided contained 60 mg of elemental iron and 0.4 mg of folic acid and was administered by teachers once weekly through a fixed-day distribution at school. This composition and regimen were adaptations agreed upon by stakeholders, as the exact WHO recommended composition was unavailable in Pakistan, and a weekly regime was chosen due to anticipated difficulties with daily adherence. The iron-folic acid (IFA) supplements were provided to the adolescent girls free of cost through their schools. To support consumption and adherence, students consumed the WIFAS tablets at school in the presence of teachers on a pre-designated day. This was typically done after recess to ensure that the girls did not take the tablets on an empty stomach. To prevent stockouts, the WIFAS supply chain management followed a structured three-phase model: centralized procurement, strategic storage at the DHA, and distribution managed by the DEA. Teachers estimated demand, submitted requisitions validated by Assistant Education Officers (AEOs), and collected supplements monthly.

The nutrition and anaemia awareness sessions were delivered by teachers, who received training. Teachers provided the girls with information on the importance of a healthy diet, the WIFAS regime, the consumption of IFA tablets, and specifically how to prevent and manage any potential side effects resulting from WIFAS consumption. The sessions also provided information on prevention and treatment of hookworm infestation through messages promoting the government’s existing deworming program in schools. The educational material used included a diet diary (*ghazai diary*) given to students to facilitate discussions about nutritious foods with their parents; flip charts and reference booklets for teachers to use during awareness sessions; and an animated story titled *Khadija’s Story*, which was screened in classrooms and broadcast on local cable TV and FM radio to extend awareness among the students and the wider community. School-based events such as speeches, quizzes, and essay writing competitions were organized to engage the students and foster mobilization.

Training was provided to a network of stakeholders considered essential for program delivery and sustainability. Teachers were positioned as primary implementation partners and change agents, while Lady Health Workers (LHWs) were also trained on technical material and community mobilization. The content of the sessions for teachers and LHWs included practical planning and provision of WIFAS, counseling techniques for consumption and side-effect management, gender-sensitive adolescent nutrition messages, and systematic monitoring and reporting procedures. The implementation strategy utilized a cascade model, beginning with Training of Trainers (ToT) to create a cadre of master trainers who then facilitated wider dissemination, ensuring consistent knowledge transfer and scalable reach. This was especially useful in the case of staff turnovers in both the health and education sectors.

## 2. Materials and Methods

A mixed-methods approach was used for this program evaluation at baseline (December 2019) and endline (January 2022). Baseline and endline cross-sectional surveys were used to assess changes in Knowledge, Attitudes, and Practices (KAP) related to anaemia prevention and WIFAS among adolescent girls. To assess acceptability and understand the perceptions of feasibility, in-depth interviews (IDIs) and focus group discussions (FGDs) were conducted with adolescent girls, mothers, LHWs, teachers, religious leaders, and government officials.

### 2.1. Quantitative Methods

A multi-stage random cluster sampling technique was used for the KAP surveys, with the selection of 50 public schools across the district as the primary sampling units and 15 school-going adolescent girls aged 10–19 years from each school as the secondary sampling units, using Probability Proportional to Population Size (PPS). A sample size of 750 adolescent girls was calculated with a statistical significance of 0.05, power of 0.80, and a design effect of 1.5. Adolescent girls were randomly selected using school attendance registers and recruited to participate at baseline and endline. At baseline, 703 girls were surveyed, while 752 girls were surveyed at endline. Quantitative data were collected from adolescent girls using a structured questionnaire. Skip patterns were used to enhance relevance; adolescent girls who reported no awareness of anaemia skipped subsequent detailed questions about their knowledge on anaemia and the symptoms, causes, effects, and prevention of anaemia. Descriptive analysis was used for quantitative data to obtain frequencies and percentages for categorical data and means for continuous variables. Chi-square tests were employed to compare changes in KAP proportions between baseline and endline. Statistical significance was defined as *p*-value < 0.05. Data were analyzed using SPSS (version 26).

Cases of anaemia averted during each phase of the program were estimated using outcome modeling by inputting the estimated anaemia prevalence for adolescent girls in Pakistan, the established effect size of weekly iron-folic acid supplements from systematic reviews [23], and coverage of adolescent girls reached with a full scheme of WIFAS at each phase of the program.

### 2.2. Qualitative Methods

The Socio-Ecological Model and the spheres of influence guided purposive sampling across the stakeholder groups. Twenty-two IDIs were conducted with a diverse range of stakeholders, including NI, implementing partners, and government officials at federal, provincial, and district-levels, as well as community leaders, LHWs, and teachers. Forty-three FGDs were conducted with adolescent girls, their parents, teachers, community members, and LHWs. Eighteen FGDs were held with adolescent girls, each with ten participants per group. Girls were further separated into two groups; one group with girls aged 10–15 years and another with girls aged 16–19 years. Sixteen FGDs were conducted with teachers, two FGDs with LHWs, and seven FGDs with community members and parents, averaging five participants per group. Participants in the IDIs and FGDs were selected using purposive sampling to ensure that diverse perspectives, experiences, and gender dynamics were captured. Qualitative data were collected using semi-structured guides. Potential bias from data collectors was mitigated by intensive training and validation of 23% of forms, which exceeded the standard 5%.

Prior to data collection, enumerators underwent two-day comprehensive field training covering study objectives, survey instruments, respondent approach techniques, ethical considerations, and informed consent procedures. The study questionnaires and semi-structured IDI and FGD guides were pre-tested to ensure the clarity, accuracy, and effectiveness of data collection procedures in a community setting.

Data from both IDIs and FGDs were collected through audio recordings, detailed notetaking by the moderator on key points and non-verbal cues, and participant observation. The qualitative data analysis followed a thematic content analysis approach. All audio recordings were transcribed verbatim in the local language and then translated into English.

The Socio-Ecological Model not only guided the qualitative sampling, but was used as a deductive framework to guide the initial coding of IDIs and FGDs. Transcripts were first coded inductively to capture emergent ideas; then, codes were mapped onto the model’s levels: individual (adolescent girls’ knowledge, attitudes, side-effect experiences), interpersonal (influence of parents, peers, and teachers), institutional (school environment, teacher training, supply chain within the education and health systems), and policy (cultural norms, religious leaders’ views, and perceived government support for scale-up). Within each stakeholder group or sphere of influence, data were coded to identify themes of acceptability, feasibility and impact. Initial themes were generated and organized within each level to explain how factors at multiple layers enabled or hindered the feasibility and acceptability of the WIFAS pilot program. Through iterative review and discussion, these themes were subsequently merged and synthesized into three overarching categories: (1) enabling environment and institutional systems; (2) community engagement and behavior change; and (3) adolescent empowerment and agency. Enabling environment and institutional systems included codes for merging sub-themes such as a multisectoral policy and advocacy foundation, institutional alignment through formal MOUs, procedural delays and resolution, pandemic resilience and teacher leadership, supply adaptation and advocacy, and a multilayered accountability system. Community engagement and behavior change comprised trust-building through direct observation, engagement of religious and community gatekeepers, school and mass media mobilization, and gender-sensitive capacity-building and awareness sessions. Adolescent empowerment and agency included a shift from misconceptions to personal benefits and emerging self-efficacy and peer advocacy (Table 2). Findings from the IDIs and FGDs were also triangulated with quantitative results to ensure consistency, accuracy, and a more complete understanding of implementation processes. For the process mapping component, the research team manually constructed a detailed descriptive account through an iterative reading of transcripts. Qualitative data were analyzed using NVIVO software (version 14).

To ensure rigor, internal coding consistency was maintained by using multiple coders to analyze transcripts and have differences reviewed by the team lead. The lead researcher checked for intra- and intercoder variability in a subsample. Analyses were then conducted for convergence and divergence within and across stakeholder groups. Credibility was enhanced by triangulating qualitative findings with quantitative data, and by independent replication of reported adherence rates in a third-party endline survey. Data collection was conducted by qualitative research experts with master’s degrees, and regular debriefing sessions supported reflexivity. Key analytical challenges included a mismatch between official school registry data and actual numbers verified during field spot-checks, COVID-19-related delays that necessitated virtual consultations, and burdensome manual registers, leading to the recommendations for digitalization.

Written informed consent was obtained from all adult participants prior to enrolment in the study. Additionally, informed assent was obtained from adolescent girls, and consent was obtained from their primary caregiver. The Institutional Review Board (IRB) of Research and Development Solutions (RADS) in Islamabad, Pakistan, granted ethical approval on 18 October 2019 for the study.

## 3. Results and Discussion

### 3.1. Quantitative Results and Discussion

Through the pilot phase of the program, WIFAS was provided to 6545 school-going adolescent girls aged 10–19 years across 50 schools over 24 weeks, with approximately 5253 girls completing a 12-week supplementation cycle. During the scale-up phases, 30,506 girls were reached with the full scheme of WIFAS in Lodhran, Swabi and Pishin (2024–2025). Based on NI’s Outcome Modelling for Nutrition Impact Tool, the pilot is estimated to have prevented 1008 cases of anaemia among adolescent girls in the first phase and 5659 cases of anaemia by scaling up to reach 30,506 adolescent girls with at least one full scheme (12+ supplements over the 6 months preceding the reporting period).

The sample size was 750 adolescent girls at each time point for the KAP survey, with completion rates ranging from 94% to 100% (Table 3). A refusal rate of 6% at baseline was observed due to a lack of parental consent and/or simple refusal. However, there were no refusals observed at endline.

Household size remained stable between baseline and endline, with a mean of seven members per household (Table 4). A minor shift in family composition was observed, with the prevalence of nuclear families decreasing from 81% to 78%, whereas joint families increased from 19% to 22%. There was a substantial change in the distribution of the head of household’s occupation. The proportion of heads of households engaged in farming on their own land declined from 34% to 24%, and an increase in daily wage workers from 26% to 32% was observed. Modest increases were also recorded in private sector employment (7% to 8%) and government employment (7% to 9%).

The age distribution of adolescent girls increased among younger girls aged 10–14 years from 76.3% to 78.8%, with a corresponding decrease in older girls aged 15–19 years. The proportion of girls having completed middle school (6–8 years) declined from 75% to 72%, while those having attained secondary or intermediate education increased from 25% to 28%. At endline, 27% of adolescent girls had access to both the internet and social media.

Nuclear families were found to be more common than joint families in our evaluation. This finding is contrary to previous evidence from Punjab, where the joint family type prevails over the nuclear family [27]. However, more recent national trends show an ongoing transition from joint to nuclear family structures in Pakistan, driven by a combination of rapid urbanization, economic shifts, and changing social values that are breaking down the traditional, multigenerational joint family system [28,29]. Regardless of family structure, female education and empowerment remain critical factors in improving both personal and familial health and well-being [30].

Due to the skip pattern used, the number of adolescent girls responding to knowledge-related questions on anaemia (*n* = 490) was lower, as only those demonstrating an awareness of anaemia were asked follow-up questions on their knowledge. Knowledge about anaemia increased significantly, with 100% of girls demonstrating awareness about anaemia compared to 68% at baseline (Table 5). Understanding the causes of anaemia also improved markedly; knowledge on how low hemoglobin can cause anaemia increased from 4% to 86%, and recognition of iron deficiency as a cause rose from 19% to 75%. Awareness of common signs and symptoms such as weakness, listlessness, and fatigue also showed significant improvements. Knowledge of preventive measures improved significantly, particularly regarding iron-folic acid supplements, which increased from 17% to 94%. Awareness that consumption of dark leafy greens can prevent anaemia had a significant increase from 67% to 94%. Knowledge of meat and egg consumption as a preventive measure to anaemia also increased, from 70% to 72% and from 11% to 27%, respectively. However, knowledge of seafood and legumes as foods that prevent anaemia remained low and showed negligible change at endline. Adolescent girls also attributed reduced weakness (i.e., low energy, fatigue, and physical debility), feeling stronger and anaemia prevention as perceived benefits of WIFAS consumption.

A pronounced shift was observed in the reported sources of knowledge on anaemia. Teachers became the predominant source of knowledge on anaemia for 98% of adolescent girls at endline compared to 36% at baseline, whereas the role of parents, peers, and health workers diminished to negligible levels. Dietary practices also showed positive changes. The proportion of girls consuming fewer than two meals per day decreased from 53% to 25%, while those consuming more than three meals per day increased from 47% to 75%. Skipping meals declined, with the prevalence of skipping breakfast and lunch decreasing from 38% to 19% and from 50% to 28%, respectively. The proportion of girls who never skip a meal increased from 3% at baseline to 46% at endline. There was a comprehensive positive shift in knowledge and self-reported dietary behaviors related to anaemia prevention among adolescent girls. Significant improvements were also observed in self-reported hygiene practices, with the practice of handwashing with soap before eating increasing from 73% to 85% and the practice after defecation increasing significantly from 29% to 81%.

Our evaluation demonstrated that structured nutrition and anaemia awareness sessions can produce a substantial transformation in both knowledge and self-reported behaviors related to anaemia among adolescent girls. Not only was an increase in general awareness of anaemia (increase of 32%) observed, but a fundamental shift in understanding specific causes, symptoms, and prevention methods was also noted, especially regarding low hemoglobin (increase of 83%), iron deficiency (increase of 56%), consumption of dark leafy greens (increase of 28%), and supplementation (increase of 78%). Improvements in knowledge among adolescent girls in the pilot program translated into reported improvements in daily practices, such as reduced meal skipping and increased meal frequency. Similar improvements in knowledge and behaviors were observed in Jordan, where a quasi-experimental study across four public secondary schools demonstrated that a one-month nutrition education program, employing interactive lectures, videos, and brochures, produced significantly higher total KAP scores among adolescent girls in the intervention group compared to the control group (*p*-value < 0.001) [31]. Studies from Rajasthan, Bangalore, and Gaza, where nutrition educational interventions were conducted from seven days to three months, also showed significant improvements in KAP scores among adolescent girls [32,33,34]. The educational interventions in India and Palestine consisted of lectures, wall writings, video, booklets, and brochures. Research from rural India and Indonesia also suggests that poor knowledge of healthy dietary practices is associated with anaemia among adolescent girls [34,35]. Our findings, along with the previously mentioned studies, emphasize the need for behavior change interventions, including nutrition education sessions, to support improvements in awareness and practices related to anaemia prevention among adolescent girls.

Another notable finding from our evaluation is the complete shift in the primary source of information on anaemia, from a mix of parents and health workers to teachers. This suggests that the intervention successfully leveraged the school system to create a consistent and effective channel for nutrition and anaemia information and messages. Studies from rural India implementing WIFAS programs for adolescents in schools also found that teachers were essential in influencing WIFAS awareness, provision, supervision and consumption [36,37,38]. Furthermore, our evaluation demonstrates that a school-based approach can successfully provide adolescent girls with knowledge and influence modifiable practices to combat anaemia, leading to perceived health benefits and better dietary habits in Pakistan.

Program coverage was universal, with 100% of adolescent girls reporting receipt of one or more supplements in the past six months (Table 6). Additionally, 98% of girls received at least 12 supplements over the past six months, while 74% received at least 24 supplements over the same period. A high level of adherence was observed, as 80% of girls consumed at least 12 supplements over the past six months—the WHO recommended WIFAS scheme. All adolescent girls reported consuming the supplements provided in school. Challenges with consumption were minimal, and only 1% of the girls reported experiencing challenges with WIFAS consumption at endline. Future intentions regarding WIFAS consumption were largely positive, with 94% intending to recommend it to others and 71% intending to continue consumption. Most adolescent girls attended nutrition and anaemia awareness sessions at school (89%), with a smaller proportion attending community sessions (17%). Furthermore, 93% of girls reported receiving messages on proper consumption and side-effect prevention of WIFAS. These findings indicate the benefits of a strong behavior change component and integrated teacher training on delivery of nutrition and anaemia messaging.

Our evaluation shows that a school-based WIFAS program can achieve high coverage and adherence among adolescent girls when service delivery is effectively integrated into teacher’s roles. Our pilot program attained universal coverage within the school and was met with high acceptability, as most adolescent girls both received and regularly consumed supplements. This success may have been possible through the robust nutrition and anaemia awareness sessions, which informed girls on proper WIFAS consumption and side-effect management, risks of anaemia and potential benefits from WIFAS. Evidence from similar settings indicates that incorporating messaging and BCC strategies is critical for enabling WIFAS adherence [39,40,41,42]. Programs offering only supplements demonstrate lower adherence, whereas those integrating nutrition messages achieve higher adherence rates in adolescents when attendance is high. Girls need to be regularly attending a school to enable regular consumption. Research studies from India and Vietnam have also shown a decrease in anaemia prevalence when adolescent girls fully adhered to the WIFAS scheme [43,44]. Direct WIFAS provision and supervision of consumption by teachers may have also contributed to compliance in our pilot program. A school-based WIFAS program in Pondicherry also found relatively high coverage among adolescents, with counseling received from trained teachers indicated as the primary contributing factor; this was similar to the findings from our evaluation [36,45]. Moreover, the positive future intentions of girls to both recommend and continue the supplements indicate that our pilot program is not only feasible but also highly acceptable. Our findings on WIFAS distribution and consumption suggest a strong potential for the program’s sustainability and scalability within similar contexts.

### 3.2. Qualitative Results and Discussion

#### 3.2.1. Creating an Enabling Environment and Institutional Alignment

Stakeholders attributed the success and feasibility of the pilot program to the use of a multisectoral approach, extensive advocacy and partnerships, and capacity-building. The MoNHSR&C National Coordinator described the development of a robust policy framework as the foundation that boosted national commitment to adolescent nutrition as a priority. Critical policy documents include the Adolescent Nutrition Strategy [25], an evidence review [46], and a Framework for Action [47], which collectively advocate for the adoption of WIFAS for adolescent girls. Institutional support was reinforced through a national Technical Advisory Group and provincial committees for adolescent nutrition, with NI serving as a member alongside UN agencies. While this enabling environment was described to have facilitated government buy-in, procedural challenges still emerged. National-level stakeholders described experiencing delays in securing a provincial Non-Objection Certificate and subsequent district-level permissions. The delays were attributed by stakeholders to an initial unfamiliarity among health officials regarding the pilot program, which temporarily hindered a rollout of program activities. Sustained advocacy, formal documentation, and a multisectoral inception meeting resolved these delays and led to a finalized operational plan that integrated the health and education departments. Stakeholders also highlighted the institutional alignment and multisectoral collaboration outlined in the formal MOUs as enabling the pilot program to be embedded within existing government infrastructure, while defining clear custodianship for the DHA and the DEA and utilizing LHWs for supportive messages to girls and their families in the community setting.

*“Usually the practice is that we engage with one department and ignore others, however, in this project, NI and we discussed the learning from our previous project (Right Start) and kept in mind the multisectoral factor that this project is targeting school going adolescent girls and for every step we decided to involve the key officers of the education department as the activities would be done through school teachers and while the health education curriculum was health department’s domain yet the implementation was primarily through teachers who had a major role, so was important to involve the principals of schools and the respective district officers.” (IRMNCH&NP Staff, Department of Health, Punjab).*

District-level stakeholders described the multilayered monitoring system used in the pilot program as a successful approach to ensure accountability, transparency, and efficient supply chain management. This system combined paper-based tools—classroom and school registers and triplicate issuance slips—with a Microsoft Excel database and a Monitoring Information System (MIS) application that integrated with the government’s District Health Information Software 2 (DHIS2) platform. Findings from the MIS application were reported by stakeholders as being regularly presented to the District and Provincial Steering Committees to ensure strong oversight, track progress, and timely resolution of challenges, such as following up with frequently absent adolescent girls.

While reflecting on supply chain management and their role in delivering and distributing WIFAS, teachers described the inter-departmental partnership, with the Health Department providing supplies and oversight and the Education Department executing delivery to schools, as an effective and replicable model for scale-up.

*“The challenge was to get the acceptance from the education and health departments and develop some coordination mechanism between them at district-level for timely support during project implementation and this was achieved by regular meetings and sensitization sessions at all levels.” (Teacher, Lodhran District, Punjab).*

Stakeholders from NI reported experiencing a significant hurdle in supporting the government with procurement of IFA for the pilot program, which was made was more challenging as WIFAS was not on the global model list of essential medicines (EML) at the time of the pilot [11]. IFA supplements (60 mg iron + 0.4 mg folic acid) and the WHO-recommended dosage (60 mg iron + 2.8 mg folic acid) for adolescents were not commercially available during the pilot program in Pakistan. To address this, a practical interim adaptation was used by MoNHSR&C stakeholders, where a locally available IFA supplement containing 60 mg of elemental iron and 0.4 mg of folic acid, instead of the recommended 2.8 mg, was procured for distribution to adolescent girls, with careful efforts to ensure no stockouts for the maternal programs and attention to addressing the concerns of parents regarding safety and suitability for adolescents. Stakeholders also described advocating for the local production of supplements for the adolescent-specific formulation.

Capacity-building was highlighted by stakeholders as another component behind the pilot program’s success, which was designed and executed through a collaborative partnership between NI and the government, notably the provincial Department of Health and the Ministry of Education. A cornerstone of this success was the incorporation of gender and culture sensitivity, informed by formative research including a Sex and Gender-Based Analysis (SGBA), into specialized nutrition training and adapted tools for teachers, and nutrition and anaemia awareness sessions for adolescent girls and their parents. Teachers emphasized that capacity-building filled a significant knowledge gap and provided them with the specific information needed to answer questions from students and parents.

*“We knew very little about this issue in adolescent girls. During the training we understood the severity of [the] issue and [that] addressing it with IFA can help improve the future reproductive life [of adolescent girls]. The [training] content … covered everything that we needed to know for addressing the questions [and concerns of adolescent girls].” (Teacher, Lodhran District, Punjab).*

Our finding that multisectoral collaboration and institutional alignment were primary drivers of feasibility is consistent with evidence from school-based WIFAS programs in Indonesia and Ethiopia, where joint instructions from health and education offices, a clear delineation of roles, and the integration of the program into existing government structures were considered critical for success [48,49]. The tension between a supportive national policy environment and local procedural barriers, such as delays in securing formal approvals due to government officials’ unfamiliarity with the program observed in our pilot program, parallels experiences in Indonesia, where several schools declined to participate despite official directives from the government, and in Ethiopia, where adolescent nutrition was often perceived as a low priority and the newly introduced WIFAS program did not receive the advocacy efforts required to improve government, community and parental acceptance. The multilayered monitoring system, a digital platform integrated into DHIS2, used in the Lodhran pilot in Pakistan ensured transparency and accountability, and its absence would have weakened oversight. This accountability gap was also identified in Ethiopia [49]. Furthermore, our pilot program’s decision to use locally available supplements containing 0.4 mg of folic acid rather than the WHO-recommended 2.8 mg formulation is consistent with the study in Ethiopia [49]. This illustrates a persistent gap between global standards and local market realities and the necessity of government-owned supply chains in South Asia and East Africa [48,49]. WIFAS has since been added to the global WHO EML and also to the national EML of Pakistan, enabling increased access [50,51]. The transformative role of capacity-building in the Lodhran pilot program is congruent with findings from Indonesia and Ethiopia [48,49]. In Indonesia, face-to-face teacher training was associated with significant improvements in school readiness and student adherence, while, in Ethiopia, training transformed teacher perceptions from viewing the program as an additional burden to becoming effective implementers. Finally, the partnership model between health and education departments was perceived by stakeholders in the Lodhran pilot program as effective and replicable, which is corroborated by the school-based delivery models used in Ethiopia and Indonesia, where the schools led implementation with technical support from health workers. These cross-contextual parallels indicate that the operational blueprint emerging from our program is not singular but rather reflects common implementation realities and proven strategies that can inform a government-led national scale-up.

#### 3.2.2. Building Community Trust and Interpersonal Engagement

Community acceptance of WIFAS and the pilot program was cultivated by stakeholders through community and school mobilization. Prior to supplementation, district-level stakeholders highlighted conducting nutrition and anaemia awareness sessions for parents and community members to address queries, dispel myths, and create acceptance. LHWs also reported proactively performing door-to-door visits to sensitize mothers on adolescent nutrition and raise awareness on the benefits of WIFAS, while religious leaders engaged in communicating supportive Islamic decrees and countering taboos.

*“We were conscious of the community engagement needs and therefore also involved [the] Union Council Polio Eradication Committee. The [Imams] … of the mosques after Jumma prayer told the people about WIFAS implementation in the area which minimized any probability of resistance.” (District Health Authority Staff, Lodhran District, Punjab).*

National stakeholders described developing a mobilization strategy, which was integral to shift community norms, build trust, and foster demand for WIFAS among adolescent girls, their families, and communities. Teachers, health staff and community leaders reported using educational materials such as an animated video titled *Khadija’s Story* (*Khadija ki kahani*), which illustrated a narrative of the improved performance of an adolescent girl in home, sports and education through WIFAS; a diet diary (*ghazai diary*) for students to encourage awareness of dietary patterns; various job aids such as flip charts for LHWs and teachers; and anaemia prevention booklets for religious leaders. Other materials used by teachers included wall charts, branded lunch boxes, comic books, bookmarks, and stickers to improve knowledge and behaviors among adolescent girls.

*“When [mobilization] strategy was developed … everyone was taken onboard. We gave ideas such as … [the] Khadija ki Kahani i.e., how by using WIFAS a tired girl [can] became successful in sports and education.” (District Health Authority Staff, Lodhran District, Punjab).*

*“We gave a diet diary to [all] students … Teachers and parents … mention[ed] that students looked at … [the diaries] and asked parents to give … [them the healthy] … the foods. The diary has been instrumental in developing the recognition that even when project finishes, they must take care of their diet.” (District Health Authority Staff, Lodhran District, Punjab).*

Teachers were positioned as primary implementation partners and change agents in the pilot program. Teachers shared in enhancing program and WIFAS acceptance in schools through a tailored communication strategy, which relied on thematic days, inter-school competitions (speeches, quizzes, essay writing, science models, and art), the use of mobilization tools such as flip charts and booklets, and in-class screenings of *Khadija’s Story*. This was identified by teachers as critical for addressing misconceptions linking supplements to contraception or infertility and encouraging more adolescents to participate in program activities.

Other reported approaches used by teachers and health staff at the district-level were in-person consumption of the supplement and a rapid-response mechanism (WhatsApp group), which enabled stakeholders to address parental concerns and/or harmful rumors related to IFA consumption.

*“Initially we faced difficulty that there were misconceptions of its relevance with family planning and infertility but then we could dispel these, our health department also got involved, we ate these tablets in front of them.” (Teacher, Lodhran District, Punjab)*

*“The doctors’ demonstration of consuming WIFAS in front of the parents was a key practice in eliminating a barrier where resistance occurred and establishing trust” (District Health Authority Staff, Lodhran District, Punjab).*

The use of mass and local media was identified by stakeholders as amplifying reach and awareness on anaemia prevention and WIFAS acceptance. *Khadija’s Story* was broadcast on four local cable TV networks and aired on FM-98 radio, reaching an estimated 650,000 viewers and listeners. Stakeholders described producing additional radio shows and public service announcements for broadcast on local radio. Furthermore, advocacy events were conducted by national-level and district-level stakeholders to broaden public awareness on anaemia and WIFAS through local and national print, electronic, and television news outlets. Positive feedback and testimonials from girls about increased feelings of energy and perceived better concentration and academic performance further sustained community acceptance and buy-in, supported by continuous monitoring to rapidly address refusals and rumors. Health staff attributed adolescent testimonials as being critical in improving trust and social acceptance and enhancing awareness of anaemia and WIFAS in the community and households.

In response to the COVID-19 pandemic, provincial-level stakeholders recounted teachers functioning as front-line leaders and problem-solvers who used online platforms and adapted the delivery of WIFAS, which was essential for the program’s survival and success.

*“Teachers played a very active role in the success of WIFAS because you did not involve them only for doing activity rather, they were involved in the planning as well. They gave major inputs in addressing the problems as they would timely inform the issues. Their role was exemplary even during the COVID days as they will schedule IFA supplementation on the day when school would open and which locality girls would be coming to school. That was a tough time but was successfully overcome due to leadership role of the teachers.” (IRMNCH&NP Staff, Department of Health, Punjab).*

Our findings on community engagement and trust-building are consistent with evidence from school-based WIFAS programs in Africa and Asia [49,52]. The involvement of multiple community gatekeepers, including religious leaders and the Union Council Polio Eradication Committee in our pilot program, is similar to the approach used in Ethiopia where school-level community days and Parent–Student–Teacher Associations assisted in addressing weak parental engagement [49]. The practice of teachers and health workers in our pilot study publicly consuming the supplements to dispel misconceptions about contraception and infertility mirrors the strategy used in Ethiopia in which female focal persons ingested the tablets themselves, a measure credited with building trust and facilitating transparent communication. The use of culturally adapted, adolescent-friendly materials such as *Khadija’s Story* and the diet diary in our program was observed as shifting social norms and generating WIFAS demand and acceptance. Similarly, the use of posters, guides for group discussion, fact sheets and brochures in Ethiopia not only improved WIFAS acceptance, but also increased the odds of adherence 14-fold among adolescent girls aged 10–19 years. Teachers adopted the role of frontline leaders who adjusted WIFAS schedules and relied on online platforms for program planning and implementation during the COVID-19 pandemic. A similar adaptive strategy was implemented in Indonesia, with interventions such as capacity-building and technical assistance for teachers modified to an online format during the pandemic [48]. This seems to be a common strategy used by WIFAS programs to remain effective and sustainable. In our pilot program, adolescent girls’ testimonials regarding improved energy, concentration, and academic performance after consuming WIFAS contributed to community acceptance. This was also observed in Ethiopia; girls who perceived the program as beneficial became active advocates for their peers [49]. Other peer-led approaches such as motivator girls and anti-anaemia squads were observed in Ethiopia and Indonesia, indicating that endogenous advocacy is a powerful mechanism for acceptance of WIFA supplementation [48,49,52]. Although missing from our current pilot program, this approach should be included to help improve community and household acceptance during scale-up.

#### 3.2.3. Enhancing Personal Knowledge, Attitudes, and Agency in Adolescent Girls

Parents and teachers revealed a marked shift in adolescent girls’ knowledge, beliefs, and personal agency over the course of the pilot program. During the initial days of the pilot, adolescent girls shared that they held on to some misconceptions, such as fears that the supplements were contraceptives, would cause infertility, or were part of a foreign family-planning agenda. Many girls reported being reluctant to take the supplements, with some mothers reinforcing the belief that they could be harmful to their daughters. However, community and school mobilization by teachers and health staff was identified by stakeholders as clarifying the purpose of WIFAS and the consequences of anaemia and improving understanding and acceptance among adolescent girls. This resulted in girls (and even their mothers) viewing the supplements and a healthy diet as tools to improve their concentration, energy, and academic performance.

*“A healthy diet can help us study better, have more energy and keep our mind focused.* *” (Adolescent Girl, Lodhran District, Punjab).*

*“After IFA use, my daughter started getting up early, do household chores, became active, took interest in studies and went to school on time.* *” (Mother of Adolescent Girl, Lodhran District, Punjab).*

The personal experiences of adolescent girls further transformed their attitudes towards WIFAS. Girls described feeling more energetic, experiencing fewer headaches, and noticing improved appetite, which motivated continued adherence. They also emphasized their willingness to recommend the supplements to peers due to their high satisfaction with WIFAS.

*“Our appetite improved after taking these tablets. We felt energetic. We ate these happily.* *” (Adolescent Girl, Lodhran District, Punjab).*

Furthermore, a shift in self-efficacy emerged among adolescent girls. Rather than remaining passive recipients, adolescent girls described becoming change agents within their families, pushing parents to provide healthier foods and advocating for the supplements. Teachers observed that this process also strengthened trust and communication, with girls increasingly confiding in them about other personal concerns. Many girls were viewed as becoming more active in sports and extracurricular activities, reflecting a broader positive change in their personal outlook and behavior.

The initial misconceptions documented among adolescent girls in our pilot program, including fears that the supplements were contraceptives or part of a foreign agenda, reflect a broader challenge observed in other school-based WIFAS programs [48,49]. In Ethiopia, limited community engagement similarly resulted in some parents preventing their daughters from participating in the program [49]. To overcome this, the Ethiopian program relied on the public consumption of supplements by health workers and teachers, resulting in increased trust, acceptance, and transparent communication—an effective approach paralleled by our pilot program.

Research highlights a similar pathway linking perceived personal benefits to sustained WIFAS adherence as observed in our program. Adolescent girls in Lodhran reported increased energy, fewer headaches, and improved appetite, outcomes that motivated continued WIFAS uptake and peer recommendation. These perceived cognitive and physical benefits align with evidence from Ethiopia linking reductions in anaemia to enhanced brain function and school performance, and to improved hemoglobin levels among girls from Indonesia [48,49]. Collectively, this evidence suggests that perceived personal improvements are central to converting initial skepticism into sustained WIFAS uptake.

#### 3.2.4. Triangulation

The mixed-methods design enabled the program’s measured outcomes to be triangulated and contextualized through the experiences of stakeholders, such as the use of community engagement for acceptance, the systematic empowerment of teachers as trusted knowledge channels, evidence-based communication strategies to address misconceptions, sustained trust building that minimized barriers to consumption, the emergence of adolescent agency that translated knowledge into improved dietary practices, and the institutional alignment that underpinned a replicable delivery platform.

The reduction in refusals to participate in the KAP interviews from 6% at baseline to 0% at endline supports the success of the mobilization and community engagement strategy described by stakeholders. Strategies included female doctors and teachers publicly consuming the supplements, Imams delivering supportive Friday sermons, and LHWs conducting door-to-door sensitization. These findings demonstrate that proactive, culturally sensitive engagement can eliminate initial resistance and secure community acceptance.

The shift in teachers as the primary source of anaemia knowledge from baseline to endline may be linked to teachers being systematically empowered through a cascade training model. Through the model, teachers reported being equipped with educational materials, which enabled them to be positioned as frontline change agents. These findings indicate that a well-trained and supported school-based platform can become a trusted and effective channel for adolescent nutrition education.

The substantial increase from 17% to 94% in knowledge of weekly iron-folic acid supplements preventing anaemia may be explained by the use of a tailored communication strategy. Stakeholders identified specific myths associated with fears of contraception and infertility being addressed through teacher-delivered awareness sessions that used culturally adapted, adolescent-friendly educational materials. This suggests that an evidence based, culturally grounded communication approach contributed to the large gains in knowledge among adolescent girls.

The low proportion of adolescent girls reporting challenges with WIFAS consumption at endline (1%) may be attributed to trust-building measures undertaken by teachers, doctors and LHWs. Teachers and health workers described using a real-time WhatsApp rapid-response group to enable the timely resolution of concerns and publicly consuming the supplements to dispel myths linked to contraception and infertility. This suggests that sustained community engagement was instrumental in minimizing barriers to WIFAS consumption among adolescent girls.

Moreover, the marked declines in adolescent girls skipping breakfast and lunch, along with the increase from 3% to 46% in girls never skipping a meal, may be partly explained by the girls’ accounts of internalizing the anaemia and nutrition messages received during awareness sessions. Girls reported requesting healthier foods at home, while teachers observed girls being empowered to join sports and extracurricular activities. These findings suggest that awareness sessions were not limited to knowledge acquisition but reflected a wider shift in personal agency.

Contrarily, the persistent, limited knowledge of seafood and legumes as anaemia-preventing foods (~6–7%) among girls may be linked to a specific content gap in the educational materials used during awareness sessions. This highlights the need for broadening dietary messaging in future programming.

The near-universal coverage and high adherence observed among adolescent girls, combined with the modeling estimates of over 5000 anaemia cases averted, can be associated with stakeholders’ accounts of a meticulously organized supply chain and inter-departmental partnership. This may represent a measurable outcome of institutional alignment and an achievable impact through a feasible and acceptable delivery platform, which led stakeholders to characterize the pilot program as effective and replicable.

#### 3.2.5. Sustainability and Scaling Up

As the first WIFAS program in Pakistan, to the best of our knowledge, this pilot has been pivotal in providing key data for decision-making to enable a potential transformation of the national adolescent nutrition landscape. The pilot’s perceived success was attributed to a multisectoral foundation, including the indispensable government partnership and institutional alignment, the leveraging of existing health and education systems to ensure ownership and sustainability and the proactive community and teacher engagement to build trust and dispel myths. This effective foundation coupled with additional external funds enabled the program’s scale-up to new districts, demonstrating a viable pathway for addressing adolescent anaemia in similar contexts through a combined strategy of supplementation, education, and systemic reinforcement. A national dissemination event shared achievements and lessons learnt while presenting an ideal platform to advocate for scale-up.

In 2022, the government launched a scale-up phase during which the program was expanded into new districts, including Swabi in Khyber Pakhtunkhwa and Pishin in Balochistan, while also extending to all tehsils within the Lodhran District. This expansion was preceded by scoping studies and a detailed mapping of schools, students, teachers, and local health infrastructure in the targeted areas. To secure institutional support, provincial sensitization meetings were held with Health and Education Departments in Khyber Pakhtunkhwa and Balochistan, and with the IRMNCH&NP in Punjab.

Prior to this program, very few national approaches on anaemia prevention existed for adolescent girls in Pakistan. Approaches have consisted primarily of IFA supplementation only being provided as part of short-term donor-funded pilots or projects [53]. Similarly, school feeding programs for adolescents have also been sporadic, with little to no focus on anaemia prevention [54,55]. This gap may be contributing to the lack of improvement observed in the national prevalence of anaemia from 2000 (40.6%) to 2019 (41.3%) among adolescent girls and women [19,56].

#### 3.2.6. Strengths and Limitations

The strengths of this study include a description of an innovative adolescent nutrition project in a context where adolescent girls have not been the focus of public health and nutrition efforts. The study used a mixed-methods design, a multi-stage sampling strategy, and multi-stakeholder engagement, which provide a nuanced assessment of the pilot’s impact on the KAP of adolescent girls, and the modeled impact of WIFAS on reducing anaemia among girls. The hemoglobin levels of adolescent girls were not assessed at pre- and post-supplementation. Some limitations include the inclusion of only school-going girls within one district of Punjab, which may restrict generalizability and limit scope, in a context where many girls do not regularly attend school. Changes may be attributed to factors other than supplementation and nutrition awareness sessions due to the lack of a control group. The use of self-reported data may introduce recall bias or social desirability bias. Follow-up studies are recommended to include large, multi-district samples and biochemical analysis of pre- and post-supplementation and nutrition education interventions.

## 4. Conclusions

This pilot program demonstrated the feasibility of a school-based WIFAS intervention to prevent anaemia among adolescent girls in a high-burden setting in a context where few nutrition and health services are available to adolescent girls. By employing a multisectoral approach that integrated health and education systems, and by embedding the intervention within existing government infrastructure, the program established a feasible delivery mechanism. Success was attributed to the creation of an enabling environment through institutional alignment, multisectoral collaboration, culturally adapted, adolescent-friendly materials, and robust community mobilization that proactively addressed socio-cultural barriers. The pilot not only provided direct nutritional benefits to thousands of girls but also generated critical implementation evidence, highlighting the importance of teacher engagement, supply chain strengthening, and digital monitoring. These findings offer a replicable blueprint for scaling up adolescent anaemia prevention across Pakistan and similar contexts to close the critical gap in adolescent health and anaemia. Financial investments will be required to sustain and expand impact. Equity in reach must be considered, as many adolescent girls with the greatest risks of malnutrition and anaemia are not regularly attending schools and are currently not reached by preventative services. Health systems must also be strengthened to be adolescent-responsive. The potential integration of WIFAS into national social protection frameworks, such as Benazir Income Support Programme’s Nashonuma program, signifies a pivotal shift towards institutionalizing adolescent nutrition within broader poverty alleviation strategies.

## Figures and Tables

**Table 1:** Nutritional indicators of adolescent girls aged 10–19 years in Pakistan, in Punjab and in the district of Lodhran [20,26].

Indicator	National	Punjab	Lodhran
Households, %	*n* = 96,307	*n* = 35,902	*n* = 741
Food secure	63.1%	67.6%	37.8%
Adolescent girls, %	*n* = 37,482	*n* = 13,313	*n*= 339
Underweight ^1^	11.8%	10.5%	10.2%
Severely underweight ^2^	3.6%	3.3%	2.0%
Overweight ^3^	16.8%	17.6%	16.1%
Obese ^4^	5.5%	5.5%	3.7%
Stunted ^5^	28.5%	26.3%	22.7%
Severely stunted ^6^	11.2%	10.2%	6.3%
Anaemia ^7^	54.7% (*n* = 14,309)	53.6% (*n* = 6368)	56.6% (*n* = 201)
Gross enrolment ratio in secondary education	50%	62%	43%

^1^ Underweight: Weight for age < −2 SD. ^2^ Severely underweight: Weight for age < −3 SD. ^3^ Overweight: Weight for age > +1 SD. ^4^ Obese: Weight for age > +2 SD. ^5^ Stunted: Height for age < −2 SD. ^6^ Severely stunted: Height for age < −3 SD. ^7^ Anaemia: Hemoglobin levels < 12 g/dL.

**Table 2:** Primary categories and themes from WIFAS pilot program in Lodhran.

Primary Category	Themes/Sub-Themes
Enabling Environment and Institutional Systems & Providers	Multisectoral Policy and Advocacy Foundation
	Institutional Alignment through Formal MOUs
	Procedural Delays and Resolution
	Pandemic Resilience and Teacher Leadership
	Supply Adaptation and Advocacy
	Multilayered Accountability System
Community Engagement and Behavior Change	Trust-Building through Direct Observation
	Engagement of Religious and Community Gatekeepers
	School and Mass Media Mobilization
	Gender-Sensitive Capacity-Building and Awareness Sessions
Adolescent Empowerment and Agency	Shift from Misconceptions to Personal Benefits
	Emerging Self-Efficacy and Peer Advocacy

**Table 3:** Survey response rate.

	Baseline			Endline		
	Target	Completed	Refusal	Target	Completed	Refusal
KAP surveys						
Adolescent girls, *n* (%)	750	703 (94%)	47	750	752 (100%)	0

**Table 4:** Socio-demographic characteristics at baseline and endline.

Socio-Demographic Characteristics	Baseline	Endline
Household	*n* = 703	*n* = 752
Persons per household, mean	7	7
Family type, %		
Nuclear family	81%	78%
Joint family ^1^	19%	22%
Occupation of head of household, %		
Farmer (own land)	34%	24%
Entrepreneur	18%	17%
Daily wage worker	26%	32%
Private sector employee	7%	8%
Government employee	7%	9%
Trader	2%	1%
Unemployed	3%	1%
Unable to work due to disability	0%	0%
Other	2%	7%
Adolescent girls		
Age in years, %		
10–14	76.3%	78.8%
15–19	23.7%	21.2%
Educational attainment, %		
Middle (6–8 years)	75%	72%
Secondary (10–11 years) + intermediate (11–12 years)	25%	28%
Access to internet, %	N/A	27%
Access to social media, %	N/A	27%

^1^ Joint family: Extended family consists usually of >3 generations and their spouses living as a single household.

**Table 5:** Knowledge, attitudes and practices of adolescent girls at baseline and endline.

	Baseline	Endline
Knowledge	*n* = 490	*n* = 752
Know about anaemia, %	68%	100% *
Know low hemoglobin can cause anaemia, %	4%	86% *
Know iron deficiency can cause anaemia, %	19%	75% *
Know weakness is a sign or symptom of anaemia, %	36%	79% *
Know listlessness is a sign or symptom of anaemia, %	23%	41% *
Know fatigue is a sign or symptom of anaemia, %	19%	34% *
Know a diet rich in micronutrients can prevent anaemia, %	29%	59% *
Know iron-rich foods can prevent anaemia, %	22%	37% *
Know consumption of meats ^1^ can prevent anaemia, %	70%	72%
Know consumption of seafood ^2^ can prevent anaemia, %	7%	6%
Know consumption of eggs can prevent anaemia, %	11%	27% *
Know consumption of legumes ^3^ can prevent anaemia, %	7%	6%
Know consumption of dark leafy greens ^4^ can prevent anaemia, %	67%	94% *
Know iron-folic acid supplements can prevent anaemia, %	17%	94% *
Source of knowledge on anaemia, %	*n* = 490	*n* = 752
Teachers	36%	98% *
Parents	34%	0% *
Peers	4%	2%
Health workers	12%	0% *
Others	14%	0% *
Dietary practices	*n* = 703	*n* = 752
Consumed <2 meals per day, %	53%	25% *
Consumed >3 meals per day, %	47%	75% *
Usually skip breakfast, %	38%	19% *
Usually skip lunch, %	50%	28% *
Usually skip dinner, %	10%	7%
Never skip a meal, %	3%	46% *
Practice of handwashing with soap, %	*n* = 703	*n* = 752
Before eating food	73%	85% *
After defecation	29%	81% *

^1^ Meats: Beef, liver, lamb, chicken, and organ meats. ^2^ Seafood: Fish (rahu, mackerel, snapper) and shellfish (shrimp, crab). ^3^ Legumes: Lentils, beans (kidney, pinto, black), chickpeas, soybeans, and peas. ^4^ Dark leafy greens: Spinach, kale, Swiss chard, and beet greens. * *p* < 0.05.

**Table 6:** WIFAS distribution and consumption of adolescent girls at endline.

	Baseline	Endline
WIFAS	*n* = 703	*n* = 752
Received >1 supplement in the past 6 months, %	0%	100% *
Received ≥24 supplements over past 6 months, %	0%	74% *
Received >12 supplements over past 6 months, %	0%	98% *
Consumed >12 supplements over past 6 months, %	0%	80% ^1,^*
Consumed WIFAS received at school, %	0%	100% *
Experienced challenges with WIFAS consumption, %	0%	1%
Intention to recommend WIFAS consumption to others, %	0%	94% *
Intention to continue WIFAS consumption in future, %	0%	71% *
Nutrition and anaemia awareness sessions		
Participated in session at school, %	0%	89% *
Participated in session in community, %	0%	17% *
Received messages on WIFAS consumption and side effects prevention, %	0%	93% *

^1^ Adherence data from data entry sheet. * *p* < 0.05.

## Data Availability

The raw data supporting the conclusions of this article will be made available by the authors on request.

## Abbreviations

The following abbreviations are used in this manuscript:

AEOs	Assistant Education Officers
DALYs	Disability-Adjusted Life Years
DEA	District Education Authority
DHA	District Health Authority
DHIS2	District Health Information Software 2
EML	Essential Medicines List
FGDs	Focus Group Discussions
GAIN	Global Alliance for Improved Nutrition
GNI	Gross National Income
IDA	Iron Deficiency Anaemia
IDIs	In-Depth Interviews
IFA	Iron-Folic Acid
IPC	Interpersonal Communication
IRB	Institutional Review Board
IRMNCH&NP	Integrated Reproductive Maternal, Newborn and Child Health and Nutrition Programme
KAP	Knowledge, Attitudes, and Practices
LHWs	Lady Health Workers
MEAs	Monitoring and Evaluation Assistants
MIS	Monitoring Information System
MoNHSR&C	Ministry of National Health Services, Regulations and Coordination
NI	Nutrition International
PPS	Probability Proportional to Population Size
RADS	Research and Development Solutions
SGBA	Sex and Gender-Based Analysis
SHNSs	School Health and Nutrition Supervisors
SPSS	Statistical Package for the Social Sciences
SUN	Scaling Up Nutrition
ToT	Training of Trainers
UN	United Nations
UNICEF	United Nations Children’s Fund
USD	United States Dollar
WHO	World Health Organization
WIFAS	Weekly Iron-Folic Acid Supplementation
WRA	Women of Reproductive Age
